# American Veterinary College

**Published:** 1885-04

**Authors:** 


### Proceedings Veterinary Medical Societies.

### AMERICAN VETERINARY COLLEGE.

The tenth annual commencement of the American Veterinary College was held at Chickering Hall, Wednesday evening, March 4th, 1885. Clark Bell, Esq., delivered an address, Prof. C. A. Doremus awarded the prizes, and Samuel Marsh, Esq., conferred the degrees upon the following graduates: Edwin M. Barnes, Theadore Birdsall, George Bowers, D.V.S., Wessales M. Brodhead, Abraham L. Brown, Robert F. Burleigh, M.D., Martin Cushing, William Dimond, George W. Dodin, Hugh F. Doris, Edward Douglas, D.V.S., William J. Elliott, Owen W. Finley, Jacob F. Folker, Christopher Horseman, Samuel H. Kent, Honore F. Laine, William R. J. Mitchell, James McCaffrey, John N. Navin, Jr., Robert M. Navin, William H. Prophett, Stephen L. Richards, Frederick P. Ruhl, Julius W. Scheibler, Philip H. Seltzer, George M. Steck, Dominick J. O'Sullivan, Alpheus A. Tuttle, John P. Wilson, Haru Taka Yokura.

---

